# Variable Density Filling Algorithm Based on Delaunay Triangulation

**DOI:** 10.3390/mi13081262

**Published:** 2022-08-05

**Authors:** Yujing Qiao, Ning Lv, Xuefeng Ouyang

**Affiliations:** 1 School of Mechanical Engineering, Yangzhou Polytechnic College, Yangzhou 225009, China; 2 School of Automation, Harbin University of Science and Technology, Harbin 150080, China

**Keywords:** 3D printing, concave polygon convex decomposition, poisson disk sampling, Delaunay triangulation, fill traces

## Abstract

The quality of the filling algorithm in additive manufacturing directly affects the strength of the part. The commonly used 3D printing filling algorithm at this stage is the classic filling algorithm. The density of each part in the filling region is the same, and there is a cavity structure in the transverse direction, which makes the strength of the part in the transverse direction lower. Therefore, this paper proposed a new filling algorithm—variable density filling algorithm based on Delaunay triangulation. First, we performed concave-polygon-convex decomposition on the filling area to form printing sub-regions; then, the filling density value was set according to the required intensity of each region, and we used the Poisson disk sampling algorithm to generate the filling point set. Finally, Delaunay triangulation was performed on the generated point set to generate filled traces. The comparison with the two commonly used classical filling algorithms proves that the algorithm can improve the strength of the part to a certain extent, and the printing time and the consumption of consumables will not increase significantly.

## 1. Introduction

3D printing technology uses modeling, layering, path planning, stack printing, and other steps to generate 3D models and make parts [1,2,3]. 3D printing parts have been widely used in aerospace, military industry and weapons, automobiles and racing cars, electronics, biomedicine, dentistry, jewelry, games, consumer goods and daily necessities, food, construction, education, and many other fields, and have become a rapidly developing trend [4,5,6,7,8]. Filling path planning is an important process link in 3D printing, the generated filling pattern directly affecting the strength of the printing model [9,10]. The commonly used filling algorithms at this stage include straight line filling and contour offset filling, etc., but there are holes in a certain direction, resulting in insufficient strength of printed parts [11,12].

In order to solve the defects of the above-mentioned filling algorithm, researchers have conducted a lot of improvement research on the filling method. Literature [13] proposed a method to automatically fill the contour obtained by 3D scanning, generate the surface from the points near the contour, and fill the contour area by generating point sequence on the surface. This method is suitable for structures filled with a large number of holes, as the point sequence interpolation of the cavity region is performed automatically without user input. Soo K. M et al. proposed the use of 3D warping technology to effectively fill the holes generated when creating a virtual-viewpoint image [14]. In order to reduce the blurring effect in the hole region filled by the conventional algorithm and to enhance the texture quality of the generated virtual view, in-painting algorithm was used, and the boundary noise in the initial virtual view obtained by 3D warping was also removed. After 3D warping, relative location of the background to the holes was estimated, and then the pixels adjacent to the background were preferentially filled by using the information of the adjacent objects to obtain better results. Furthermore, when searching for the most similar patches, the temporal inconsistency between frames can be reduced by extending the search region up to the previous frame. Zhang et al. proposed a novel filling algorithm, which can realize the connection of sub-regions by using Fermat spiral on the basis of continuous filling, and integrates convex decomposition, PCA, variable spacing filling, and other operations, further improving the printing efficiency [15]. Jin et al. proposed an approach to parallel filling path generation based on adaptive gaps for fused deposition printers [16]. The proposed tool-path generation method first determines the inclination of the reference line according to the lower number of sharp corners in the paths. Then, the filling area is divided into three stages. In the pre-sweeping stage, a group of parallel lines with predetermined gap and optimized inclination are used to intersect the filling boundary and store the intersection points between them. The second stage adjusts some sweeping line segments which satisfy several specific conditions, and the last stage divides the filling areas into several sub-regions according to the selected sweeping lines. Subsequently, the sweeping lines in each sub area are adjusted separately to adapt to the uniform gap. Finally, some adjacent sub-paths are connected to reduce the number of uncut paths. Other methods, such as using machine learning algorithm to realize adaptive filling, are still in the experimental stage, and the prediction accuracy of the model cannot fully meet the actual machining requirements. 

The above-mentioned filling methods of 3D printing are based on the existing filling path planning algorithms, and use various methods to compensate the printing cavity to improve the printing intensity, but none of them have generalization, and the degree of industrial practicality is weak. Literature [12] draws the following conclusions by comprehensively comparing the mechanical properties of various filling schemes: the 2D infill patterns outperformed the tested 3D infill patterns on both strength-to-weight ratio and peak load. Among the 2D infill patterns that performed well, Grid, Cross, and Triangles seem to be the best patterns to choose in order to have a light part that will support one-dimensional compressive loading. Overall, infill densities of 80% or 100% are more efficient than lesser ones in terms of strength-to-weight ratio. Depending on the situation, when a desired part does not have the need to be as light as possible, it would be recommended to use a high infill density or even print it in full. However, it is obvious that high-density filling increases the cost of printing and the weight of the printing part. Therefore, in this study, we proposed a novel filling algorithm, which uses Delaunay triangulation net as the filling pattern from the filling mechanism to enhance the strength of the printing part.

Our method can set the filling density according to the required strength of each printing region. Combined with the filling pattern of the triangulation network, it can make the stress on all segments of the printing part more uniform, and will not significantly increase the printing consumables. Therefore, our print filling method not only improves the overall load strength of the printing part, but also has strong industrial practicability, and can also meet individual needs well.

## 2. Partitioning and Convex Decomposition of Filling Regions

Since the Delaunay triangulation network needs to be constructed within convex polygons, illegal triangles will be generated if the Delaunay triangulation network is constructed within concave polygons [17], so the filling regions need to be divided first, and convex decomposition performed on the concave polygon [18].

In additive manufacturing, STL files are stored in the form of triangular patches. After the layering slices are completed, the set of contour line information points for each layer will be obtained, in which the outer contours are arranged counterclockwise. When the known contour is counterclockwise, it is only necessary to judge whether each point is a concave point. The vector formula of $p_{i}$ and its adjacent two points $p_{i-1}$ and $p_{i+1}$ on the contour are $\vec{P_{i-1}P_{i}}$ and $\vec{P_{i}P_{i+1}}$. By calculating the value of $\left|\vec{P_{i-1}P_{i}}\times \vec{P_{i}P_{i+1}}\right|$, we can determine whether point $p_{i}$ is a concave point. Let the coordinates of the three points $p_{i-1}$, $p_{i}$ and $p_{i+1}$ be $\left(x_{i-1},y_{i-1}\right)$,$\left(x_{i},y_{i}\right)$ and $\left(x_{i+1},y_{i+1}\right)$, respectively, then the vector expression formed by these three points is Formula (1).

(1) $$\left\{\begin{array}{c} \vec{P_{i-1}P_{i}}=(x_{i}-x_{i-1},y_{i}-y_{i-1})\\ \vec{P_{i}P_{i+1}}=(x_{i+1}-x_{i},y_{i+1}-y_{i}) \end{array}\right.$$

When calculating the concave point, the vector cross product formula need to be used. The cross-product formula of any two vectors $\vec{\;a}$ and $\vec{\;b}$ is as Formula (2).

(2) $$\vec{\;a}\times \vec{\;b}=\left|\begin{array}{ccc} i & j & k\\ x_{a} & y_{a} & z_{a}\\ x_{b} & y_{b} & z_{b} \end{array}\right|$$

Let $z_{a}=z_{b}=0$ and substitute $\vec{P_{i-1}P_{i}}\;\vec{P_{i}P_{i+1}}$ into Formula (2) to obtain Formula (3).

(3) $$\vec{P_{i-1}P_{i}}\times \vec{P_{i}P_{i+1}}=\left(x_{i}-x_{i-1}\right)\times \left(y_{i+1}-y_{i}\right)-\left(x_{i+1}-x_{i}\right)\times \left(y_{i}-y_{i-1}\right)$$

The positive and negative of Formula (3) are used to judge the concavity and convexity of points, which can be classified into the following three situations:

(1) When $|\vec{P_{i-1}P_{i}}\times \vec{P_{i}P_{i+1}}|>0$, the point $p_{i}$ is a convex point.
(2) When $|\vec{P_{i-1}P_{i}}\times \vec{P_{i}P_{i+1}}|=0$, the three points are collinear.
(3) When $|\vec{P_{i-1}P_{i}}\times \vec{P_{i}P_{i+1}}|<0$, the point $p_{i}$ is a concave point.

According to the above method, the infilled regions can be decomposed into one or more convex polygons to facilitate Delaunay triangulation.

## 3. Selection of Filling Point Set and Determination of Density

The method in this paper needs to generate random point sets first, and then perform Delaunay triangulation on the random point sets. Path planning and infilling are performed according to the grid generated by Delaunay triangulation. In the process of generating random points, if pseudo-random numbers are used to generate random points, the random seed is determined by the system clock. Therefore, when the algorithm is determined, the random seeds are certain, and the generated random points are basically fixed, which violates the original design intention of random point selection. Additionally, the generated point set has a low randomness, uneven distribution of random points, and poor infill effect. In view of this, Poisson-disk sampling is used to select random points to solve the problem of uneven distribution of random sampling points [19].

**Figure 1:**
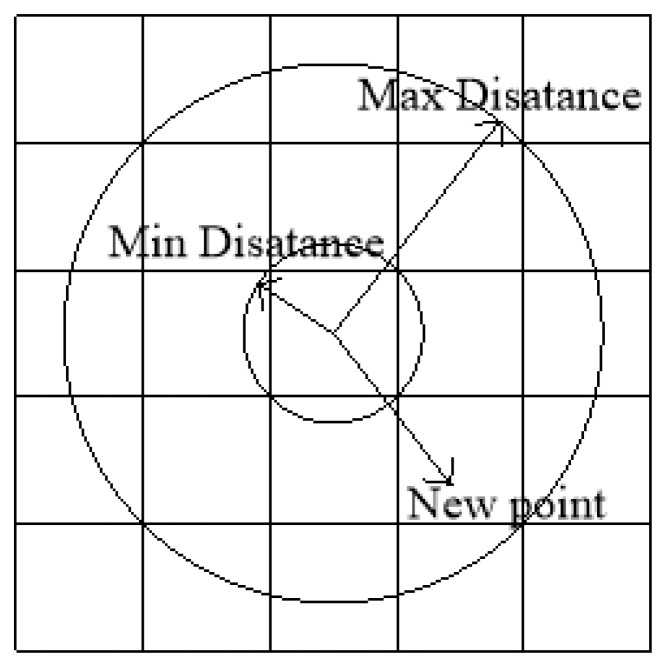
Poisson-disk sampling.

The input of Poisson-disk sampling includes the sampling region D, the minimum radius *r* of disk sampling and the constant *k* as the input, where the disk sampling radius determines the density of the point set, and the filling density is also changed by changing the disk sampling radius, *k* is the sample limit to be selected before rejecting in the algorithm, and *k* = 30 is usually selected. The specific process of sampling is as follows:

(1) Initialize a grid; each cell stores at most one sampling disk point for storing samples and accelerating spatial search. The diagonal length of the cell is the radius *r* of the sampling disk, which ensures that no matter where the sampling disk is in the cell, the cell can be covered. The application of cell technology means that only the points in the 5 × 5 grid need to be considered when calculating the distance from the new point to the existing point, greatly reducing the amount of calculation.
(2) Randomly select a sampling point, insert it into the sampling region grid, and initialize the active list with the sampling location stored at that index.
(3) When the activity list is not empty, a random index is selected from it, and at most *k* points are uniformly selected from the annular region around point xi with radius *r* and 2*r*, as shown in Figure 1. Check if the 5 × 5 range where these points lie is within distance *r* of the existing samples. If a point is far enough from an existing sample point, it is taken as the next sample point and added to the active list. If no such point is found after *k* attempts, the point xi is removed from the active list.

The density of the points needs to be set when selecting points in the filling point set, and the filling density is controlled by setting the density of the points. Since points in the filling point set are selected by Poisson-disk sampling, set the sampling radius of Poisson disk. The filling density is the ratio of the filling traces area to the total area of the outline frame, and its expression is as shown in Formula (4).

(4) $$\rho =\frac{S^{\prime }}{S}$$

where, *S*’ is the area of the filling traces, and *S* is the total area surrounded by the contour line. Formula (5) is used to calculate the area of the filling traces:

(5) $$S^{\prime }=l\times d$$

where: *l* is the total path length of filling traces, and *d* is the width of filling trace. The unified trace width used in this experiment is 1.2 mm.

In this paper, when using Delaunay triangulation for filling, it is necessary to first determine the radius of Poisson selected points, and then perform Delaunay triangulation on the selected point set. The filling path can only be calculated after triangulation, and the meshes of each Delaunay triangulation are different. Through experiments, we found that when the radius of the Poisson-disk was set at 2 mm, the filling density was about 100%; when the sampling radius of the Poisson-disk was set at 4 mm, the filling density was about 50%; and when the sampling radius of Poisson-disk was set at 6 mm, the filling density was about 35%. In this way, through the inversion of many experiments, we obtained the relationship between the filling density and the radius as shown in Formula (6).

(6) $$\rho =\frac{2}{r}$$

**Figure 2:**
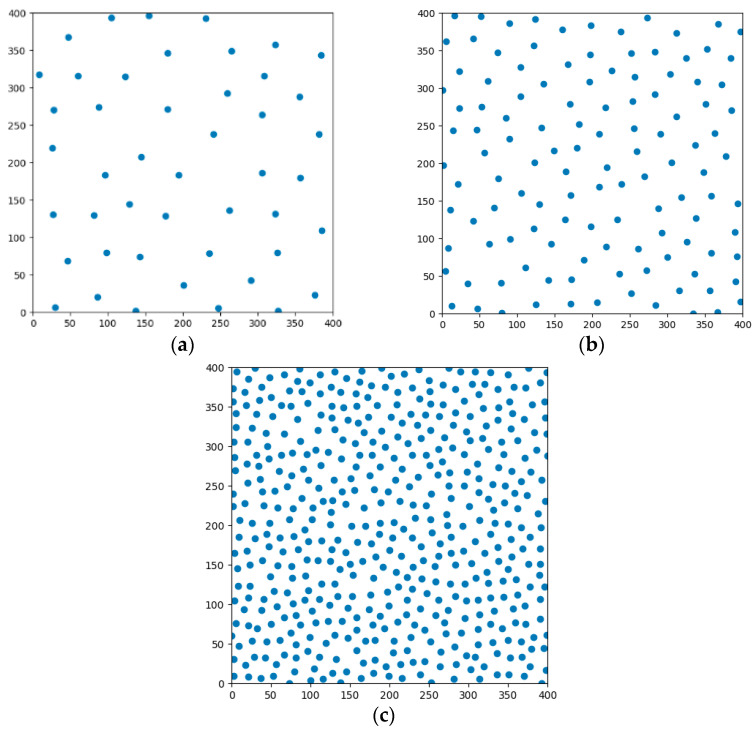
Poisson disk sampling. (**a**) 10% fill density; (**b**) 40% fill density; (**c**) 80% fill density.

We performed many experiments, compared Formula (4) with Formula (6), and verified that the density error difference between the two formulas was within 5%, so Formula (6) was used to set the filling density, and point sets selected with different radii are shown in Figure 2.

Figure 2 shows a simulation diagram of using Python to implement point selection. Points are selected in a 400 × 400 square region. The filling densities in Figure 2a–c are 10%, 40%, and 80%, respectively. It can be observed that the distribution of point sets was uniform, and there was no uneven distribution of randomly selected points. In the actual filling process, when variable density is used for filling, it is only necessary to set the filling density of each area respectively before selecting points, so that the effect of different densities of different printing areas on the same layer can be achieved.

## 4. The Network Construction Algorithm Based on Delaunay Triangulation

### 4.1. Local Optimization Procedure (LOP) Algorithm

**Figure 3:**
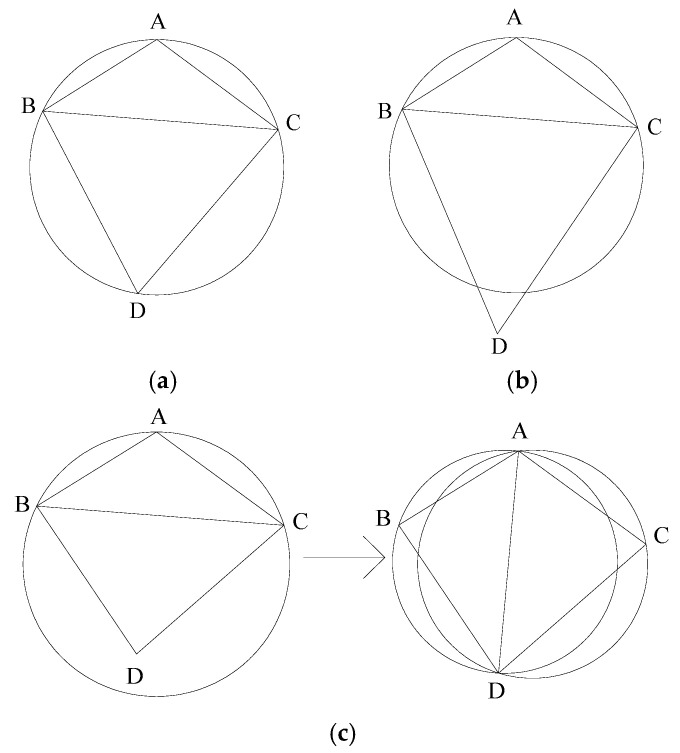
The relationship between point *D* and circumcircle of triangle ABC. (**a**) Point *D* is on the circumcircle; (**b**) Point *D* is outside the circumcircle; (**c**) Point *D* is inside the circumcircle.

When generating the Delaunay triangulation, some triangles that do not conform to the properties of the Delaunay triangulation are often generated [20], and the LOP algorithm is required for adjustment and optimization. The LOP algorithm is a local algorithm proposed by Lawson, which is used to judge whether the points outside the Delaunay triangulation are within its circumscribed circle [21]. It is judged according to the principle that the sum of opposite angles of cyclic quadrilateral is 180°. There are mainly the following three situations:

(1) When sin(∠A+∠D)=0, the point *D* is just on the circumcircle of triangle ABC, and the diagonals do not need to be exchanged at this time, as shown in Figure 3a.
(2) When sin(∠A+∠D)>0, point *D* is just outside the circumcircle of triangle ABC, and the diagonals do not need to be exchanged at this time, as shown in Figure 3b.
(3) When sin(∠A+∠D)<0, the point *D* is just inside the circumcircle of the triangle ABC, and the diagonals need to be exchanged, as shown in Figure 3c.

In the process of generating triangulation net, due to the large number of generated triangulation net, and the point set of the triangulation also being randomly generated, certain errors cannot be avoided. Therefore, it is necessary to use the LOP optimization algorithm, which can make the triangulation network fully meet the property requirements of Delaunay triangulation. 

### 4.2. Improved Network Construction Algorithm of Delaunay Triangulation

**Figure 4:**
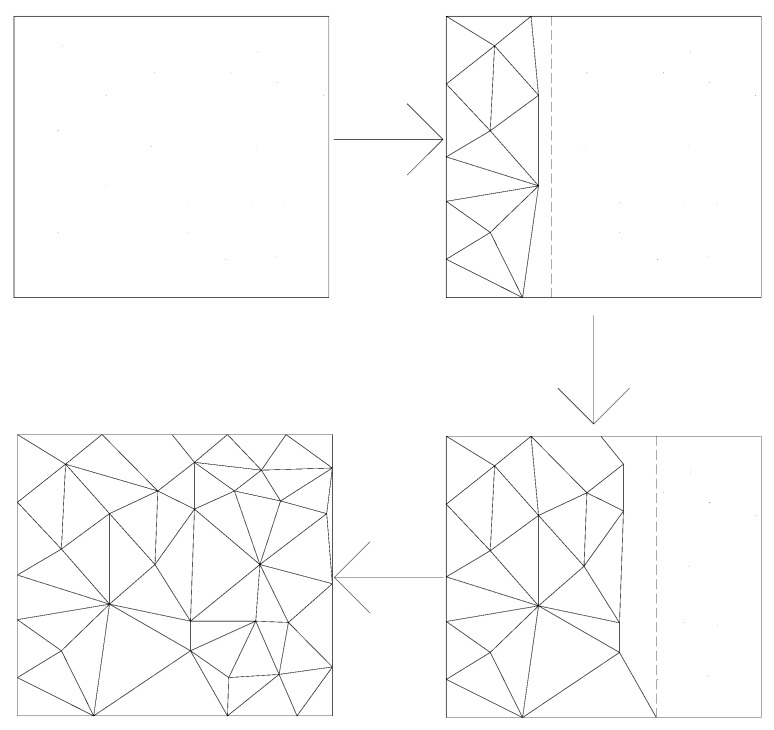
Improved Delaunay triangulation algorithm.

When constructing Delaunay triangulation network and filling triangulation network, although the calculation of the triangulation net growth algorithm is simple, the calculation amount is large and time-consuming [22]. The point-by-point insertion algorithm is slow in constructing a triangular network, and it is only suitable for convex polygons [23]. The divide-and-conquer algorithm mainly has high hardware requirements and also requires a large number of recursive algorithms, resulting in low running speed [24]. Based on the above points, our design optimizes and improves the construction algorithm of the Delaunay triangulation network. The main idea of the improved algorithm is to form a triangular network in parallel and segment by segment, as shown in Figure 4. The specific process is as follows.

(1) The point set *S* generated by Poisson-disk sampling is bubble sorted by the abscissa and stored in the linked list A.
(2) The $X_{max}-X_{min}$ in linked list A is calculated, and then stores it to facilitate the next step to construct the Delaunay triangulation by segment by segment.
(3) Gradually advance from *X_min_* to *X_max_* when generating Delaunay triangulation. First, connect the polylines in the Y-axis direction that are closer to *X_min,_* and then find the points that are closest to each segment of the polyline in the point set to form a triangulation net. Then use the newly generated two sides as the base edge to find the closest point, and gradually advance to generate triangulation until all points in the current region form a triangulation.
(4) When the triangulation of the previous region is completed, the irregular polyline close to the division line of the previous region is taken as the reference edge to construct the Delaunay triangulation of the next region until the triangulation of all regions is completed.
(5) Perform LOP optimization on the Delaunay triangulation to ensure that each triangle conforms to the properties of the Delaunay triangulation.

The test found that the improved Delaunay triangulation method has a better effect in generating Delaunay triangulation network, because it uses the parallel propulsion network to generate Delaunay triangulation, with faster generation speed, less computation, and less hardware requirements.

### 4.3. Implementation of Variable Density Filling Algorithm Based on Delaunay Triangulation

**Figure 5:**
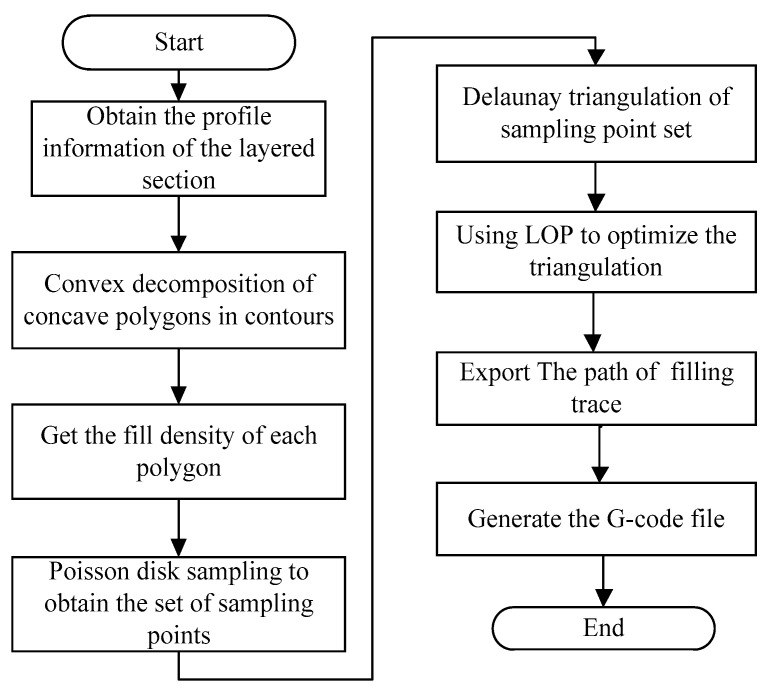
The flow chart of filling algorithm.

In the filling process, the printed parts may require different strengths in different regions, but the classical filling algorithm can only fill with the same density. Our algorithm can preset the filling region density of different filling region, and select points in different filling regions with different Poisson sampling radii; after the concave polygon is convexly decomposed, each sub-convex polygon is filled with the required density value so as to achieve different filling density effect in different regions. The specific flow of the algorithm is as follows:

(1) The information points of the contour are derived, and the contour is segmented by using the convex decomposition algorithm of concave polygons to obtain multiple sub convex polygons, and then each convex polygon is encoded.
(2) The preset filling density of each region, that is, the Poisson disk sampling radius, samples and selects points in the convex polygon obtained in step (1).
(3) Delaunay triangulation is performed on the point set selected in step (2) to generate a triangulation network.
(4) The triangulation generated in step (3) is optimized by using the LOP optimization algorithm so that the obtained triangulation conforms to the properties of the Delaunay triangulation.
(5) The Delaunay triangulation obtained after optimization is the filling path, which is exported to the G-code file for printing. The filling algorithm flow is shown in Figure 5.

## 5. Experimental Research and Analysis

Based on the open-source code of Cura Engine, it was developed using Visual Studio2017 (Microsoft Corp, Redmond, WA, USA) and Pycharm2020 (JetBrains, Prague, Czech Republic) platforms, and the algorithm was implemented using C++ language and Python language joint debugging, and then embedded in the FDM-3D printer control system for algorithm performance testing. The experiment used an XYZ FDM-3D machine, the printing model material was PLA plastic, different filling methods were used, and the same printing parameters were used. The printing process parameters and values of the experiment are shown in Table 1.

Two groups of models were selected for the test simulation. One model uses the H-shaped bracket for the strength test, and the other group models uses the five-point star as the complex polygon to test and study whether there will be problems in the filling of complex polygons. At the same time, five-point star model was used for the variable density filling algorithm based on Delaunay triangulation.

**Figure 6:**
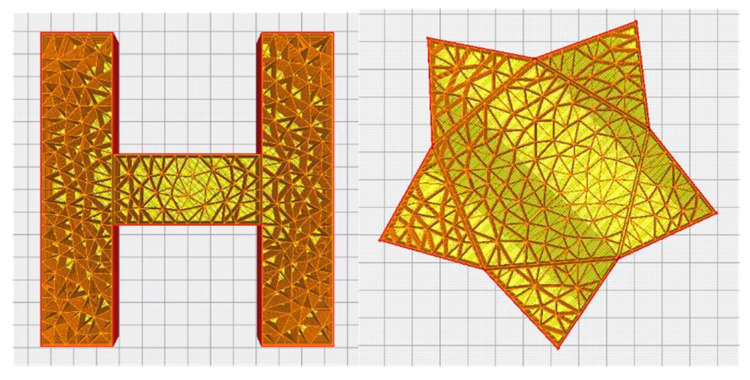
Cura simulation model.

The slice simulation of the two test models based on the Delaunay triangulation filling algorithm is shown in Figure 6. It can be observed that the filling based on the Delaunay triangulation filling algorithm did not appear to be too dense or too sparse, and the filling was uniform.

**Figure 7:**
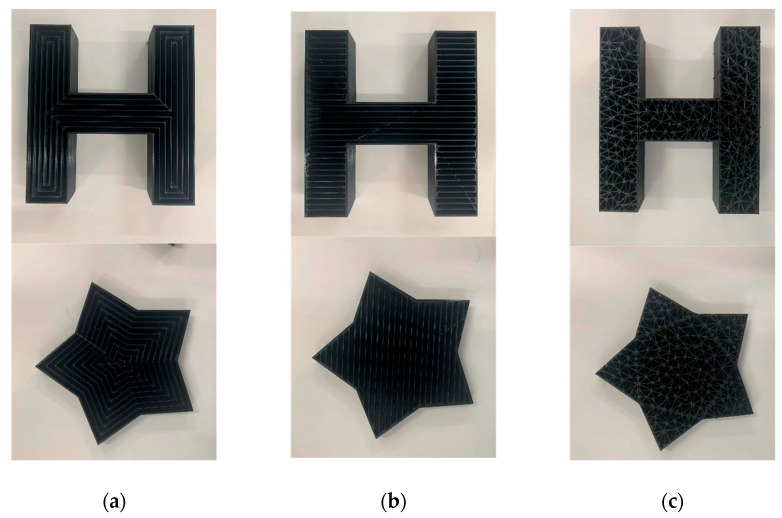
Printed physical objects. (**a**) Contour offset filling; (**b**) straight lines filling; (**c**) Delaunay triangulation filling.

The comparison analysis and verification between Delaunay triangulation filling algorithm and classical filling method (contour bias filling and linear filling) were conducted. The test was conducted at 30% filling density respectively. Different filling algorithms were used to generate G-code files. The G-code files were imported into the 3D printing control system for the printing test. The printed finished products are shown in Figure 7.

**Figure 8:**
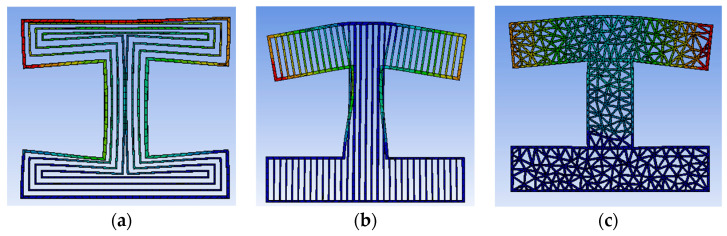
Finite element simulation diagram. (**a**) Contour offset filling; (**b**) straight lines filling; (**c**) Delaunay triangulation filling.

Figure 7a shows the model entity picture filled with contour offset filling, Figure 7b shows the model entity picture filled with straight lines filling, and Figure 7c shows the model entity picture filled with Delaunay triangulation filling. Through the observation of the physical model, it was found that there was a cavity structure in a certain direction for contour offset filling and linear filling, so the force in this direction was relatively weak. However, the filling algorithm based on the Delaunay triangulation had filling traces in all directions, and the filling also tended to be more uniform. In this experiment, reverse engineering was performed on the G-code code file generated after model slicing, reverse generating the STL model file, and then the format of STL model file was converted for finite element analysis to test the stress strength in each direction of the model under three filling densities. Firstly, the lateral stress analysis was carried out on the H-type bracket. The unit of network division was 5 mm. The outer side of one long side of the H-shaped bracket was used as the stress surface for fixation, and then the uniformly increased pressure was applied to the other long side of the H-type bracket. The pressure step was 20 Pa, and the maximum pressure was 200 Pa. A total of 10 steps of pressure were used for stress analysis. Finite element simulation using ANSYS software is shown in Figure 8. The shape after slicing with offset filling is shown in Figure 8a, the shape after slicing with linear filling is shown in Figure 8b, and the shape after slicing with Delaunay triangle filling is shown in Figure 8c.

It can be seen from the transverse pressure simulation diagram that the deformation of the model filled with the Delaunay triangulation network was the smallest, while the deformation of the model filled with straight line filling and offset filling was more serious due to its cavity structure, so the model filled with Delaunay triangulation had higher stress intensity. The displacement changes of each model under pressure are shown in Table 2.

**Figure 9:**
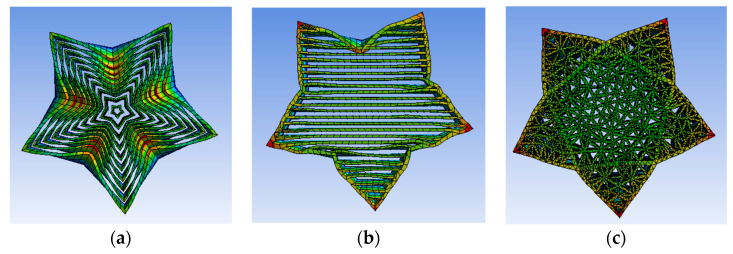
Finite element simulation diagram. (**a**) Contour offset filling; (**b**) straight lines filling; (**c**) Delaunay triangulation filling.

After the transverse test of the filling, the longitudinal test was carried out, that is, the section test was carried out for it. A more complex five-pointed star model was used to test, and tests the stress conditions of the three kinds of filling were under the same pressure conditions as the previous experiment. The finite element simulation is shown in Figure 9.

It can be seen from the longitudinal pressure simulation diagram that the deformation of the model filled with Delaunay triangulation network was still the smallest, while the deformation of offset filling and linear filling was more serious. From the force cloud diagram, it can be seen that the filling algorithm based on Delaunay triangulation network had uniform stress, which conforms to the original design. It can be seen from the stress cloud diagram that the filling algorithm based on the Delaunay triangulation was uniformly stressed, which is in line with the original intention of the design. The deformation displacement of each model after pressure is shown in Table 3. By analyzing the deformation displacement, it can be seen that the displacement distance of the filling algorithm based on Delaunay triangulation network was the smallest, so the model based on Delaunay triangulation network filling algorithm had higher stress strength.

The comparison of printing consumables and printing time is shown in Table 4. Compared with the experimental data, the results showed that when different filling methods were used for printing, our method consumed a little more consumable material and took a little longer to print. However, the filling algorithm based on Delaunay triangulation obtained better strength effects, and the model did not appear to have precision distortion. Therefore, the filling algorithm based on Delaunay triangulation has a better printing effect and is more practical in actual production.

**Figure 10:**
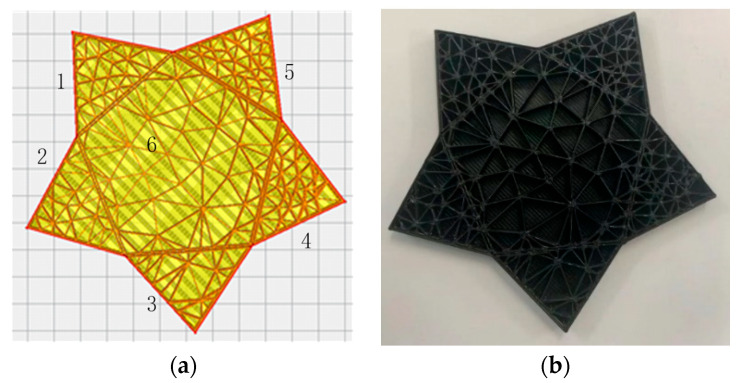
Variable density filling experiment based on Delaunay triangulation. (**a**) Filling software simulation, where the regions of 1 and 2 are 30% filling density, the region 3 is 20% filling density, the regions of 4 and 5 are 50% filling density, the region 6 is 10% filling density; (**b**) printing entity based on variable density filling algorithm.

When the variable density filling algorithm was used for filling, in order to observe the density change of each region, the five-point star model was still used. Firstly, the five pointed star model was decomposed into five triangles and a central Pentagon by concave-polygon-convex decomposition, and then the filling density value of each filling region was set, that is, the sampling radius of Poisson disk sampling was set to achieve the effect of different filling density according to the sampling radius of each region. Finally, Delaunay triangulation was performed on the set of points in each region to generate the filling traces. The filling results are shown in Figure 10. Figure 10a is the filling software simulation in which region 1 and region 2 use 30% filling density, region 3 uses 20% filling density, region 4 and region 5 use 50% filling density, and region 6 uses 10% filling density. Figure 10b shows the printing entity of the variable density filling algorithm. By observing the software simulation diagram and the physical printing diagram, we can see that using a variety of filling densities can ensure the uniformity of filling, and the printed parts also have good effects. The variable density filling algorithm is finally realized.

## 6. Conclusions

When the traditional filling method is used for filling with slicing software, the printing intensity is poor, and the longitudinal force is often better and the transverse force is weak. However, the filling algorithm based on Delaunay triangulation network cannot only makes up for the above shortcomings, but also increase the filling density for weak regions, making the strength of the parts better. In addition, as we all know, in the process of practical application, the load on each region of the part is different. Our printing method can set different printing densities according to the distribution of the load of the part, so that the force on the component is more uniform in the process of use, so as to obtain a better application effect. In terms of printing time and consumption of consumable material, the filling algorithm based on Delaunay triangulation network proposed in this paper did not significantly increase the printing time compared to the classical filling algorithm, and did not significantly increase the consumption of consumable material. Moreover, higher-strength printed parts were obtained. However, the variable density filling algorithm studied in this paper was researched in an ideal state. The actual printing process is deformed due to machine accuracy problems, and the printed parts are deformed due to temperature field changes during the extrusion process of the extrusion head. These reasons mean the deviation is not fully considered, which needs to be comprehensively considered and eliminated in engineering practice.

We will further study the segmentation of the filling regions, and set the density value of each filling region according to the wishes of the designer and the stress state in the actual use process, so as to improve the practicality and personalized setting of 3D printing. Therefore, the variable density filling algorithm based on Delaunay triangulation has a very wide range of potential practicality, and the print results will be more personalized.

## Figures and Tables

**Table 1:** Basic parameters of filling and printing.

The Printing Parameters	Parameter Values
Extrusion head temperature	190 °C
The nozzle diameter	0.6 mm
Wall thickness	1.2 mm
The width of filling line	1.2 mm
Filling density	30%
Printing speed	50 mm/s
The no-load moving speed	60 mm/s

**Table 2:** Longitudinal displacement change table.

Step Number	Offset Displacement (m)	Linear Displacement (m)	Delaunay Triangulation Displacement (m)
1	$1.3333\times 10^{-4}$	$4.9738\times 10^{-6}$	$3.8611\times 10^{-8}$
2	$2.6666\times 10^{-4}$	$9.9476\times 10^{-6}$	$7.7223\times 10^{-8}$
3	$3.9999\times 10^{-4}$	$1.4921\times 10^{-5}$	$1.1583\times 10^{-8}$
4	$5.3332\times 10^{-4}$	$1.9895e\times 10^{-5}$	$1.5445\times 10^{-7}$
5	$6.6665\times 10^{-4}$	$2.4869\times 10^{-5}$	$1.9306\times 10^{-7}$
6	$7.9998\times 10^{-4}$	$2.9843e\times 10^{-5}$	$2.3167\times 10^{-7}$
7	$9.3331\times 10^{-4}$	$3.4817\times 10^{-5}$	$2.7028\times 10^{-7}$
8	$1.0666\times 10^{-3}$	$3.9790\times 10^{-5}$	$3.0889\times 10^{-7}$
9	$1.2000\times 10^{-3}$	$4.4764\times 10^{-5}$	$3.4750\times 10^{-7}$
10	$1.2900\times 10^{-3}$	$4.9738\times 10^{-5}$	$3.8611\times 10^{-7}$

**Table 3:** Transverse displacement change table.

Step Number	Offset Displacement (m)	Linear Displacement (m)	Delaunay Triangulation Displacement (m)
1	$2.7501\times 10^{-10}$	$2.2978\times 10^{-10}$	$2.2083\times 10^{-10}$
2	$6.1878\times 10^{-10}$	$5.1700\times 10^{-10}$	$4.9686\times 10^{-10}$
3	$9.6254\times 10^{-10}$	$8.0422\times 10^{-10}$	$7.7289\times 10^{-10}$
4	$1.3063\times 10^{-9}$	$1.0914\times 10^{-9}$	$9.9371\times 10^{-10}$
5	$1.6501\times 10^{-9}$	$1.3787\times 10^{-9}$	$1.3250\times 10^{-9}$
6	$1.9938\times 10^{-9}$	$1.6659\times 10^{-9}$	$1.6010\times 10^{-9}$
7	$2.3376\times 10^{-9}$	$1.9531\times 10^{-9}$	$1.8770\times 10^{-9}$
8	$2.6814\times 10^{-9}$	$2.2403\times 10^{-9}$	$2.1530\times 10^{-9}$
9	$3.0251\times 10^{-9}$	$2.5276\times 10^{-9}$	$2.4291\times 10^{-9}$
10	$3.3689\times 10^{-9}$	$2.8148\times 10^{-9}$	$2.7051\times 10^{-9}$

**Table 4:** Three kinds of filling data: comparison table.

Models	Filling Modes	Consumption of Consumable Material (g)	Printing Time (min)
H-type bracket	Contour offset filling	83	184
	Straight lines filling	89	197
	Delaunay triangulation filling	94	213
Five-point star	Contour offset filling	21	75
	Straight lines filling	23	80
	Delaunay triangulation filling	27	87

## Data Availability

Data can be requested from the corresponding author.
